# Large B-Cell Lymphomas in the 5th Edition of the WHO-Classification of Haematolymphoid Neoplasms—Updated Classification and New Concepts

**DOI:** 10.3390/cancers15082285

**Published:** 2023-04-13

**Authors:** Katrin S. Kurz, Michaela Ott, Sabrina Kalmbach, Sophia Steinlein, Claudia Kalla, Heike Horn, German Ott, Annette M. Staiger

**Affiliations:** 1 Department of Clinical Pathology, Robert-Bosch-Krankenhaus, 70376 Stuttgart, Germany; 2 Department of Pathology, Marienhospital, 70199 Stuttgart, Germany; 3 Dr. Margarete Fischer-Bosch-Institute of Clinical Pharmacology, 70376 Stuttgart, Germany

**Keywords:** large B-cell lymphoma, diffuse large B-cell lymphoma, classification, genetics, WHO-HAEM5

## Abstract

**Simple Summary:**

This paper provides an overview of the classification of large B-cell lymphomas in the 5th edition of the WHO classification of haematolymphoid tumors due to be published in 2023 and discusses new concepts, new entities, and the relevance of new genetic findings for the classification.

**Abstract:**

The family/class of the large B-cell lymphomas (LBCL) in the 5th edition of the World Health Organization (WHO) classification of haematolymphoid tumors (WHO-HAEM5) features only a few major changes as compared to the 4th edition. In most entities, there are only subtle changes, many of them only representing some minor modifications in diagnostic terms. Major changes have been made in the diffuse large B-cell lymphomas (DLBCL)/high-grade B-cell lymphomas (HGBL) associated with *MYC* and *BCL2* and/or *BCL6* rearrangements. This category now consists of *MYC* and *BCL2* rearranged cases exclusively, while the *MYC/BCL6* double hit lymphomas now constitute genetic subtypes of DLBCL, not otherwise specified (NOS) or of HGBL, NOS. Other major changes are the conceptual merger of lymphomas arising in immune-privileged sites and the description of LBCL arising in the setting of immune dysregulation/deficiency. In addition, novel findings concerning underlying biological mechanisms in the pathogenesis of the different entities are provided.

## 1. Introduction

**Figure 1:**
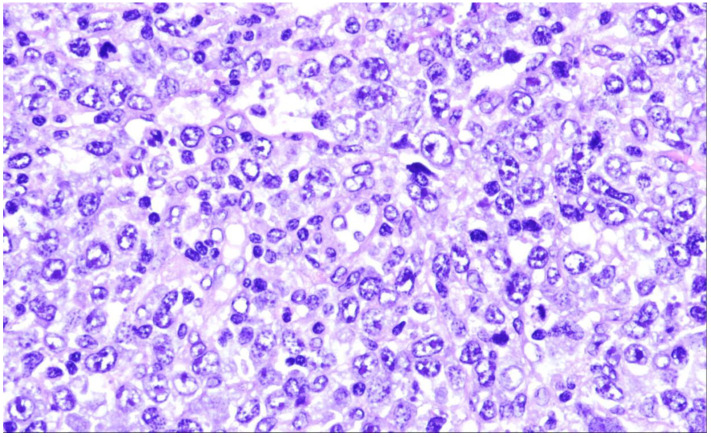
Diffuse large B-cell lymphoma, NOS (H&E ×400). There is a diffuse effacement of the architecture by medium-sized and large blastic cells that have a moderately basophilic cytoplasm and round nuclei with light chromatin and one to several nucleoli.

The family of large B-cell lymphomas (LBCL) is a heterogeneous class of tumors characterized by large lymphoid cells of the B-cell lineage that by definition form sheets or clusters. “Large cell” cytology is usually defined as a lymphoid cell with a nucleus that is larger than that of a macrophage or twice the size of a normal lymphocyte (Figure 1). Diffuse large B-cell lymphomas constitute the most common type of Non-Hodgkin lymphoma in the Western world, comprising 30–40% of cases with slight over-representation of the male gender [1,2,3]. The updated 5th edition of the World Health Organization (WHO) classification of haematolymphoid tumors (WHO-HAEM5), is currently available in a pre-published version [4] and online as a beta version and is expected to appear in print in summer 2023. It recognizes 17 specific entities of LBCL that can roughly be subdivided by morphology, genetic features, viral association, site of origin, and other, mostly overlapping, criteria (Table 1). The heterogeneous biology of this lymphoma family is reflected in the marked variability of clinical outcomes. In spite of the increasing complexity of the classification, displaying substantial biological differences between entities, rather monolithic chemotherapy regimens such as CHOP or related regimens combined with anti-CD20 antibodies still represent the standard of care in patients with symptomatic disease. Although a high proportion of patients achieve complete remission, therapy resistance or relapse still occurs in 30–40% of patients, thus evidencing an unmet need for the stratification of therapy in LBCL [2].

## 2. Diffuse Large B-Cell Lymphoma, Not Otherwise Specified

The etiology of DLBCL is multifaceted, and most likely several factors contribute to pathogenesis, in particular, genetic predisposition, immunodeficiency, and viral infection [5]. The large majority of diffuse large B-cell lymphoma, not otherwise specified (DLBCL, NOS), are of primary nodal origin and lack the criteria to be classified as one of the more specific entities. They most commonly involve lymph nodes but may also arise in extranodal lymphatic tissues such as in the tonsil or the Peyer patches of the gastrointestinal tract, or in extralymphatic tissues throughout the body [1,6]. Tumors with LBCL morphology may represent transformed disease from an indolent/small B-cell lymphoma, such as chronic lymphocytic leukemia, marginal zone lymphoma, or follicular lymphoma, or from nodular lymphocyte predominant Hodgkin lymphoma. However, the large majority of these cases arise de novo. WHO-HAEM5, for the first time, includes a separate chapter on “transformations of indolent lymphomas” specifically devoted to the clinical features, morphological traits, and genetic changes involved in the transformation.

The cytology of DLBCL can be variable. WHO-HAEM5 recognizes three morphological subtypes: centroblastic, immunoblastic, and anaplastic. Gene expression profiling (GEP) studies have identified two major profiles that can subdivide DLBCL, NOS into molecular subtypes named germinal center B-cell-like (GCB-like) and activated B-cell-like (ABC-like) with the former mainly expressing genes that are also expressed in cells in the reactive germinal center (GC), and the latter having a GEP of activated B-cells (DLBCL classification according to the cell of origin—COO) [7]. This concept was also retained in the proposal of the International Consensus Classification (ICC) [8]. The majority of studies revealed an association of the ABC-subtype with poorer clinical outcomes [9], while some did not observe a clear survival difference between patients with ABC-like and GCB-like DLBCL [10]. In order to enable molecular subtyping according to the COO classification independent of more sophisticated GEP methods, immunohistochemical algorithms continue to be allowed as surrogate markers, dividing DLBCL into GCB-types and non-GCB-types, for example by using the Hans algorithm [11,12]. Besides the GE-based molecular subtypes, DLBCL, NOS also comprise a genetic subtype, namely DLBCL, NOS with dual *MYC* and *BCL6*-rearrangements (DLBCL, NOS-*MYC/BCL6*)-see below.

## 3. The Molecular Pathogenesis of DLBCL

**Figure 2:**
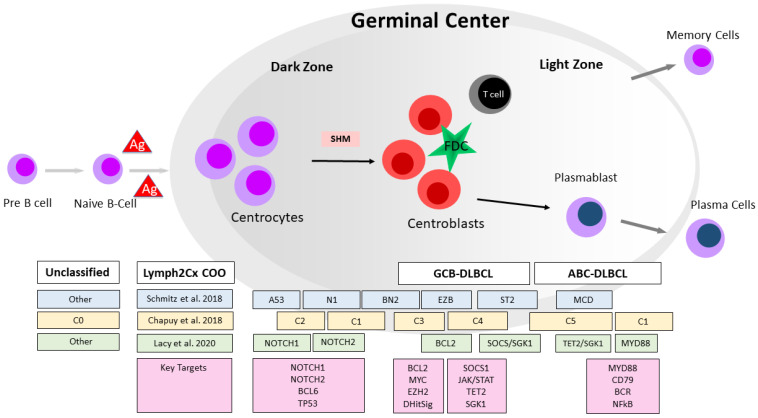
Schematic presentation of maturating and differentiating pathways in germinal centers including some biological characteristics of GCB-like and ABC-like lymphomas derived from different compartments of the GC. The lower panel shows the more recently proposed algorithms in the molecular subclassification of DLBCL including their main defining genes.

As already mentioned, DLBCL attempt to recapitulate, or rather, hijack the mechanisms of differentiation and maturation effect in GCs (Figure 2). The different molecular subtypes defined by GEP (GCB-like and ABC-like) also feature different mutational landscapes. GCB-like DLBCLs commonly show mutations in genes essential for GC development, dark zone (DZ) and light zone (LZ) transitions, and interactions with the microenvironment, such as in *EZH2*, *GNA13*, *MEF2B*, *KMT2D*, *TNFRSF14*, *B2M*, and *CREBBP* [13]. Moreover, 5–10% of GCB-DLBCL harbor dual rearrangements of *MYC* and *BCL2* genes (with or without additional *BCL6* translocations) [14,15] which, however, although morphologically indistinguishable from bona fide DLBCL, NOS, are separately classified as diffuse large B-cell lymphoma with *MYC* and *BCL2* rearrangements (see below). The large majority of DLBCL, NOS with an ABC-like GEP show a late GC/GC exit or post-GC origin and are characterized by constitutive activation of B-cell receptor (BCR) signaling and Nuclear Factor Kappa B (NFkB) pathway activation. In contrast to their GCB counterparts, they are usually negative for GC markers, such as CD10 and BCL6, but often express IRF4/MUM [11]. Upon mutation analysis, they often bear mutations in components of the BCR signaling pathway, such as in *MYD88, CD79B, PIM1,* and *PRDM1/BLIMP1* [13]. The t(14;18)(q32; q21) translocation leading to *IGH::BCL2* fusion is found in 20–30% of GCB-like DLBCL [16]. *BCL6*, on the other hand, is predominantly rearranged (30%) in ABC-like DLBCL [17]. Structural alterations of the *MYC* gene can be found in both GCB-like as well as ABC-like DLBCL [18], and often deregulate *MYC* through IG gene enhancer involvement. Around 60% of *MYC* translocations occur with an IGH, IGK, or IGL partner, while in 40% of cases, *MYC* is rearranged with a non-IG gene, including for example *BCL6, BCL11A*, *IKZF1*, or *PAX5* [19].

In *IGH::MYC* fusions, the breakpoints are usually upstream (5′) of *MYC* or within exon 1 or intron 1, but often downstream of *MYC* (3′) in non-IGH translocations [20]. Aggressive lymphomas with simultaneous rearrangements of *MYC* and *BCL6* are considered a genetic subtype of DLBCL, NOS, and in around 30% of these cases, *MYC* is directly translocated to *BCL6* forming a *BCL6::MYC* fusion as a result of the t(3;8)(q27;q24) [21]. The significance of this fusion and the difference between cases with dual *MYC* and *BCL6* translocations have not been elucidated so far.

More recently, more sophisticated approaches aiming at deciphering the mutational landscape of DLBCL, NOS via exome or targeted sequencing have shown that the molecular spectrum of DLBCL, NOS is highly heterogeneous with recurrent mutations found in approximately 150 coding genes or targets of copy number changes, and a mean of 8% of genes mutated per case [22]. Based on those findings, three groups have independently described molecular DLBCL subtypes that either further subdivide the COO classification or that are COO-independent (Figure 2, lower panel). [23,24,25]. Remarkably, in spite of the different platforms and algorithms used, these studies have provided classification trees that were overlapping, though not identical. However, since a unifying concept for the proposed clusters and their significant genetic drivers is still lacking, both WHO-HAEM5 and ICC do not yet recommend molecular subclassification of DLBCL at the present time.

## 4. T-Cell/Histiocyte-Rich Large B-Cell Lymphoma

T-cell/histiocyte-rich large B-cell lymphoma (THRLBCL) is an LBCL with <10% neoplastic large B-cells in a background rich in T-cells and histiocytes, showing some overlap with certain variants of nodular lymphocyte predominant Hodgkin lymphoma/nodular lymphocyte predominant B-cell lymphoma (NLPHL/NLPBL). No major changes have occurred as compared to the definition in WHO-HAEM4R.

THRLBCL affects lymph nodes, the spleen, the liver, and bone marrow [26]. It is a rare lymphoma usually showing a diffuse or sometimes vaguely nodular growth pattern with the previously mentioned predominating background of T-cells and histiocytic cells and randomly dispersed large B-cells that can have the appearance of peripheral B-blasts or can be Hodgkin- and Reed–Sternberg-like. There can be marked overlap with pattern E of NLPHL/NLPBL according to Fan and colleagues [27], and the distinction is usually not possible in cytologic or fine-needle preparations.

THRLBCL harbors recurrent mutations in *JUNB*, *DUSP2*, *SGK1*, *SOCS1*, and *CREBBP* genes, and these gene mutations are also encountered in NLPHL/NLPBL, which supports a possible biological relationship between the two entities [28,29]. The intriguing composition of the background is obviously due to a tolerogenic host microenvironment with a prominent pro-inflammatory and Interferon-dependent GE signature including metal-binding proteins, such as MT2A [30,31,32,33]. Among the T-cells in the background, CD4+ T-cells predominate [34,35]. Of importance, cases that contain unequivocal areas of NLPHL—and if they contain only one nodule—should be classified as NLPHL variants according to WHO-HAEM5.

## 5. Diffuse Large B-Cell Lymphoma/High-Grade B-Cell Lymphoma with *MYC* and *BCL2* Rearrangements

**Figure 3:**
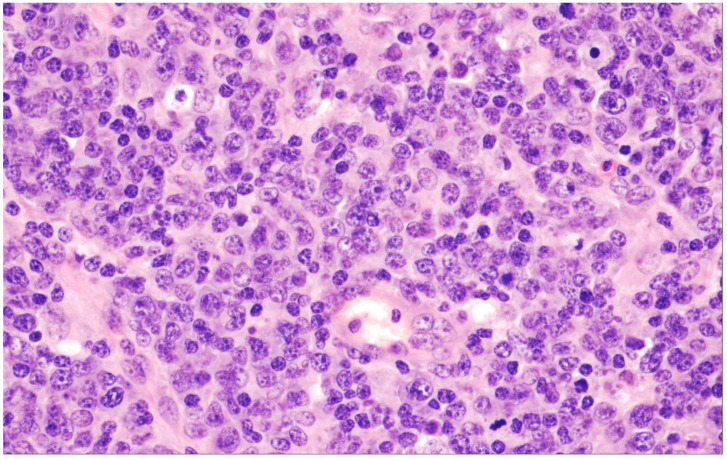
Diffuse large B-cell-lymphoma/High-grade-B-cell-lymphoma with *MYC* and *BCL2* rearrangements (H&E ×400). This tumor has a DLBCL morphology. Upon FISH, it showed a “triple hit” constellation with *MYC, BCL2*, and *BCL6* rearrangements.

The revised 4th edition of the WHO classification of haematolymphoid tumors (WHO-HAEM4R) introduced a category of “High-grade B-cell lymphoma, unclassifiable, with rearrangements of *MYC* and *BCL2,* and/or *BCL6*”, taking into account the inferior prognosis of neoplasms characterized by such dual or triple *MYC*, *BCL2,* and/or *BCL6* rearrangements. Two important changes regarding the definition and terminology of this entity were made in WHO-HAEM5. Owing to the morphological variability of these neoplasms that can be composed of large B-cells, intermediate or blastoid cells (Figure 3), WHO-HAEM5 changed the name to diffuse large B-cell lymphoma/high-grade B-cell lymphoma (DLBCL/HGBL) so that there is no need to change the diagnostic category once FISH results have been obtained. The ICC prefers the term “high-grade B-cell lymphoma”. Both in WHO-HAEM5 and the ICC, however, a fundamental change is that this entity comprises only cases with *MYC* and *BCL2* rearrangements (DLBCL/HGBL-*MYC*/*BCL2*), with or without additional *BCL6* breaks, so that aggressive lymphomas with dual rearrangements of *MYC* and *BCL6* (without *BCL2*) are excluded. The reason for this is that aggressive lymphomas with a *MYC/BCL2* double hit form a homogeneous group with respect to GEPs and the mutational profile, whereas *MYC/BCL6* DH lymphomas are heterogeneous in nature [36].

DLBCL/HGBL-*MYC/BCL2* are aggressive neoplasms diagnosed in advanced disease stages in the majority of patients. Often, there is an extranodal disease, such as infiltration of the bone marrow and the central nervous system (CNS) [37,38]. *MYC* rearrangements have been reported in roughly 10% of cases with DLBCL morphology, of which 40% were DLBCL/HGBL-*MYC/BCL2* and 10–15% DLBCL/HGBL-*MYC/BCL2* with additional *BCL6* rearrangements [18,39]. Aggressive lymphomas with an *MYC* and *BCL6* double hit and either DLBCL or HGBL morphology comprise 5 bis 10% of cases [18,39,40,41]. With the sole exception of GCB derivation, there is no adjunct marker to reliably predict cases with *MYC* and *BCL2* rearrangements. DLBCL/HGBL-*MYC/BCL2* have a GC GEP, and/or a GCB immunophenotype in 95% of cases [11,42,43,44]. Accordingly, the COO of DLBCL/HGBL-*MYC/BCL2* is a mature somatically mutated GC cell. In around 30% of the cases, there is a history of preceding follicular lymphoma (FL) [37,40,45,46,47].

In DLBCL/HGBL-*MYC/BCL2*, *IGH::BCL2* fusions are the rule [20,24,40], and *MYC* is translocated to IG loci in 60% of the cases or to non-IG partners including *BCL6, PAX5, RFTN1* and others [18,19,20,21,24]. In contrast to the rare precursor cell (lymphoblastic) neoplasms with *MYC* or dual *MYC* and *BCL2* translocations, the *MYC* translocation in DLBCL/HGBL-*MYC/BCL2* obviously arises on the constraints of a pre-existing *BCL2* translocation and, according to the structure of the IG breakpoints, takes place in the GC. DLBCL/HGBL-*MYC/BCL2* usually harbors complex karyotypic changes. The genetic landscape is characterized by mutations in genes typically altered in FL, such as *BCL2, KMT2D*, *CREBBP*, *EZH2*, and *TNFRSF14,* as well as in genes typically mutated in Burkitt lymphomas (BLs), such as *MYC*, *CCND3*, *FOXO1*, and others [46] and, therefore, differs from DLBCL, NOS as well as from FL and BL. As a consequence, most cases of DLBCL/HGBL-*MYC/BCL2* are categorized into DLBCL molecular subgroups EZB, C3, or BCL2 [23,24,25,48]. More recently, two groups have independently published GE signatures that recognize DLBCL/HGBL-*MYC/BCL2*, termed “molecular high-grade” and “double hit” signatures. Of interest, these signatures identify not only aggressive lymphomas with this double hit status but also a significant number of lymphomas that do not harbor *MYC* and *BCL2* rearrangements, although in some of them, retrospectively, cryptic rearrangements have been detected [14,15,49,50]. The fact that BLs also express these signatures indicates that they are not recognizing the double hit status per se, but a common biology related to the dark zone of the GC.

Rare cases of aggressive non-precursor B-cell lymphomas with either large cell or high-grade morphology and expression of Terminal Deoxynucleotidyl Transferase (TdT) have been described [51,52]. Of importance, such cases—although overall very rare—cluster within DLBCL/HGBL-*MYC/BCL2*, so staining for TdT is advised. Equally rare bona fide B-cell lymphoblastic leukemias/lymphomas with TdT expression and *MYC* translocations or, even rarer, *MYC/BCL2* double hit have been mainly reported in the pediatric or adolescent setting [53,54] and are molecularly different from DLBCL/HGBL-*MYC/BCL2*. More recently, flow cytometry or immunohistochemistry-based classifiers have been proposed to differentiate between the two conditions [55,56].

**Figure 4:**
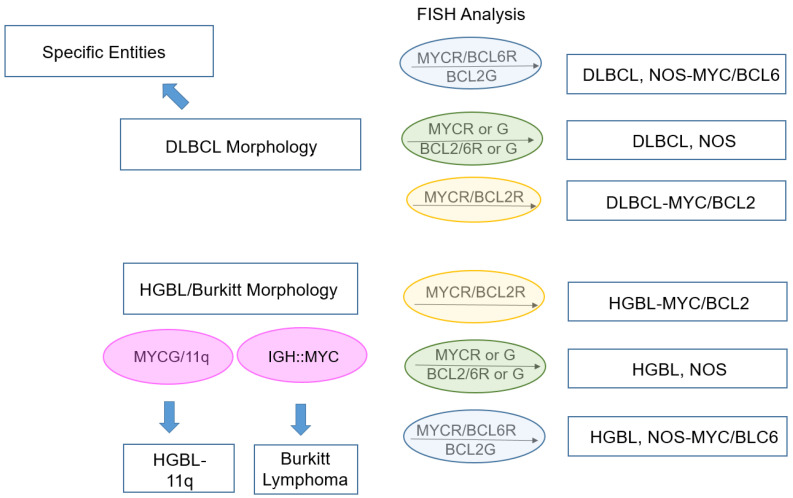
Algorithm for the classification of aggressive B-cell lymphomas in WHO-HAEM5 according to rearrangements of *MYC*, *BCL2*, and *BCL6* and/or presence of the complex 11q gain/loss patterns.

In contrast to DLBCL/HGBL-*MYC/BCL2,* aggressive lymphomas with either large cell or high-grade morphology and an *MYC/BCL6* double hit, but lacking *BCL2* translocations, form a heterogeneous tumor category without indications of a unifying biology. They infrequently show mutations of epigenetic regulators, but instead mutations in genes that are frequently altered in ABC-DLBCL, and they often display an ABC-like GEP [24,46]. WHO-HAEM5 recommends classifying them as genetic subtypes of either DLBCL, NOS, or HGBL, NOS according to their morphological features. The ICC lists them as a provisional entity in order to allow for their more precise definition and clarification of the implication of their diagnosis in the future. Of note, both FL with dual *MYC* and *BCL2* rearrangements as well as other specific large B-cell lymphoma entities with a dual *MYC/BCL2* translocation status are excluded from this category. Figure 4 gives an overview of the algorithms for the classification of aggressive B-cell lymphomas in WHO-HAEM5 depending on the rearrangements of *MYC, BCL2*, and *BCL6* and the complex 11q gain/loss pattern.

## 6. ALK-Positive Large B-Cell Lymphoma

Anaplastic lymphoma kinase-positive (ALK+) large B-cell lymphoma (LBCL) is a rare, aggressive large cell lymphoma with immunoblastic or plasmablastic morphology of the tumor cells [57] and plasmablastic immunophenotype with reactivity for plasma cell-associated markers. No major changes have been made from WHO-HAEM4R. The ALK protein is expressed in different patterns according to the genetic aberration targeting *ALK* and can be seen in the cytoplasm only or show both cytoplasmic and nuclear reactivity. In most cases of ALK+ LBCL, there is activation and phosphorylation of *STAT3*, and MYC is also often expressed [58,59,60,61]. The *ALK* gene is involved in a variety of rearrangements including the most frequent t(2;17)(p23;q23)/*CLTC::ALK* or the t(2;5)(p23;q35)/*NPM1::ALK* fusion, the latter being the only one that leads to cytoplasmic and nuclear ALK expression [60,62,63,64,65,66].

## 7. *IRF4*-Rearranged Large B-Cell Lymphoma

In WHO-HAEM4R and the ICC, *IRF4*-rearranged LBCL (*IRF4*-LBCL) is listed as a variant of FL. However, owing to its predominating large cell cytology, WHO-HAEM5 categorizes *IRF4*-LBCL as a subtype of DLBCL. *IRF4*-LBCL is characterized by rearrangements involving chromosomal band 6p25.3, the locus of the *IRF4* gene, usually in the form of an *IGH::IRF4* fusion [67,68]. This neoplasm arises in children and young adolescents, but can also be seen in older adults. The most common site of origin is the tonsils, but head and neck lymph nodes are also commonly involved. Most patients are diagnosed in clinical stage I [67,68,69,70,71,72].

*IRF4*-LBCL can display follicular, follicular and diffuse, or diffuse growth patterns and expresses, next to pan B-cell antigens, CD10, BCL2, and BCL6. Owing to the fusion, the *IRF4* gene product MUM1 is strongly and uniformly expressed. More recently, characteristic genetic alterations have been described in *IRF4*-LBCL with mutations in *IRF4, CD79b, CARD11*, and *MYD88,* chromosomal losses at 11q and 17p, and gains of chromosome 7 [67,71,73].

## 8. High-Grade B-Cell Lymphoma with 11q Aberrations

WHO-HAEM4R had listed a provisional entity of “Burkitt-like lymphoma with 11q aberrations” that now has been renamed and upgraded to a definite entity in both WHO-HAEM5 and the ICC as High-grade B-cell lymphoma with 11q aberrations (HGBL-11q) or Large B-cell lymphoma with 11q aberrations, respectively. It is defined as an aggressive B-cell lymphoma harboring a characteristic 11q gain/loss pattern without *MYC* rearrangement. These tumors often present as a localized nodal or extranodal disease in the head and neck region or in the gastrointestinal tract [74,75]. Some cases have been seen in the post-transplant setting [76]. HGBL-11q is a rare lymphoma predominantly diagnosed in children or adolescents.

**Figure 5:**
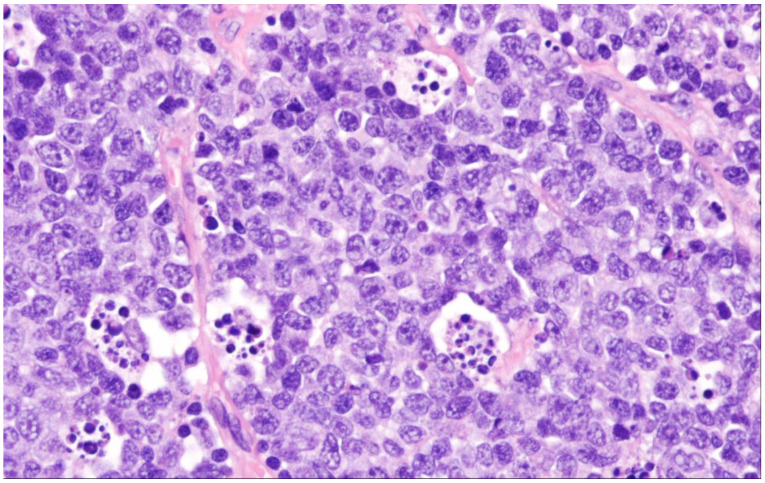
High-grade B-cell lymphoma with 11q aberrations (H&E ×400). These tumors as a rule are composed of medium-sized blastic cells reminiscent of, but more pleomorphic than, those of Burkitt lymphoma. Note the starry sky pattern with coarse cellular debris in the macrophages.

HGBL-11q characteristically shows diffuse infiltrates of cohesive medium-sized cells with a BL-like or intermediate/blastoid appearance and commonly a starry sky pattern [77]. Compared with BL the tumor cells are more pleomorphic, and the starry sky macrophages have ingested coarse apoptotic debris in 50–100% of cases (Figure 5) [77,78]. The immunophenotype resembles that of BL with reactivity for CD10 and BCL6 and negativity for BCL2, and there is a high proliferation index of >90%.

In contrast with BL, however, HGBL-11q shows a complex aberration pattern in the long arm of chromosome 11 with a consensus region of gain at 11q23.3 and a consensus region of loss at 11q24.1-qter. Rare cases harbor only the telomeric loss or telomeric loss of heterozygosity [77]. NGS analyses have shown an absence of mutations in genes typically altered in BL, such as *ID3, TCF3, SMARCA4*, or *CCND3*, while mutations in genes typically altered in GCB-DLBCL, such as *GNA13* in 50% are the rule, further substantiating the distinctiveness of HGBL-11q from BL and the rationale for changing the name [75,79].

## 9. Lymphomatoid Granulomatosis

**Figure 6:**
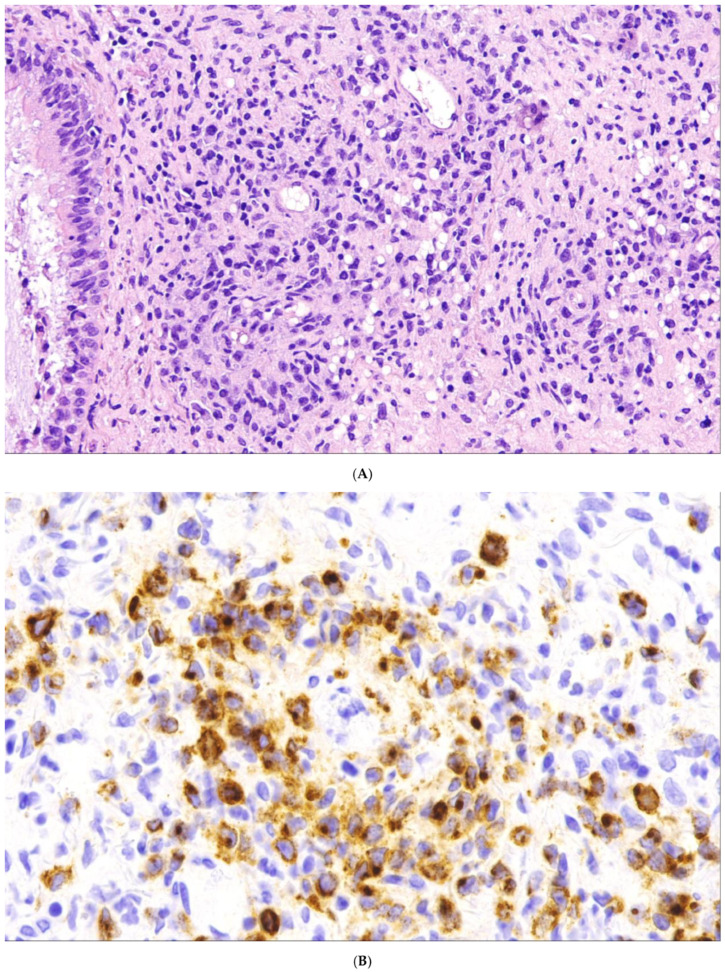
Lymphomatoid granulomatosis. (**A**) (H&E ×200). There is an angiocentric infiltration of several medium-sized blood vessels by a polymorphic lymphomatous infiltrate. (**B**) (×400) LMP staining confirms EBV association and highlights medium-sized to large blasts infected by EBV.

Lymphomatoid granulomatosis (LYG) is an angiocentric and angiodestructive B-cell lymphoproliferative disorder involving extranodal sites, which is associated with Epstein–Barr Virus (EBV) infection (Figure 6). The definition and the diagnostic criteria of LYG have not substantially changed from WHO-HAEM4R. LYG usually involves the lung and frequently the CNS, skin, kidneys, and other organs, such as the liver. In contrast, lymph nodes and bone marrow involvement are rare [80,81,82].

LYG is thought to arise on a background of impaired immune surveillance of EBV- infected cells and/or an abnormal immune response to EBV [83,84,85], however, an overt immunodeficiency state should be absent in typical cases (see below). Monoclonal IG gene rearrangements are usually identified in grade 2 and grade 3 lesions. In the setting of inborn or acquired immunodeficiency and/or dysregulation (IDD), cases with an LYG-like picture should be diagnosed according to the guidelines given in the IDD chapter in WHO-HAEM5, such as “polymorphic lymphoproliferative disorder, LYG-type, EBV+”.

## 10. EBV-Positive Diffuse Large B-Cell Lymphoma

EBV-positive DLBCL (EBV-DLBCL), now lacking the suffix “NOS” in WHO-HAEM5, is an LBCL in which the majority of the neoplastic cells harbor EBV. Since any threshold given is arbitrary, WHO-HAEM5 avoids giving a defined threshold of the number of infected cells to put a DLBCL into this entity and states that the majority of tumor cells should be EBER positive. Of note, underlying established immune deficiency or dysregulation aside from immune senescence excludes the diagnosis. EBV-DLBCL has first been described in elderly patients, thus relating it to age-related immune impairment. However, the neoplasm can occur in younger patients as well and therefore, age is not a defining criterion anymore [86].

The neoplasm is more frequent in countries with a higher EBV prevalence, such as in Asia and Latin America. It is a primarily nodal disease, however, extranodal manifestations are common, with or without lymphadenopathy [87,88,89,90,91,92].

**Figure 7:**
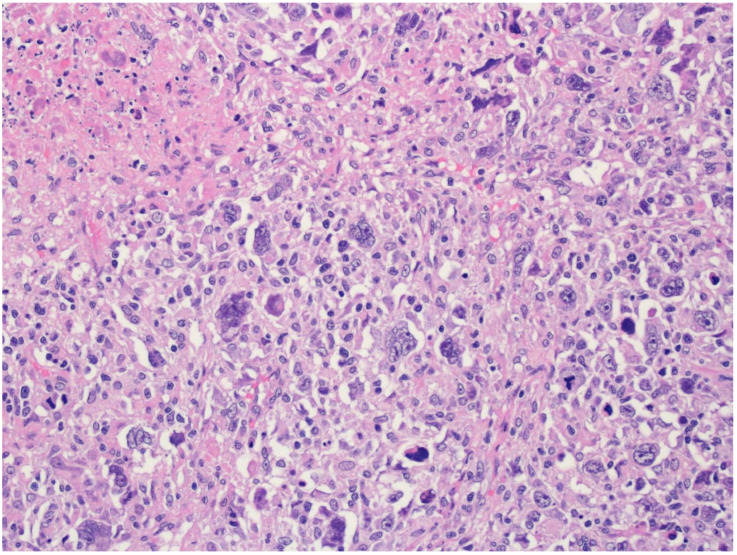
EBV-positive diffuse large B-cell lymphoma (H&E ×400). This lymphoid tumor shows numerous anaplastic Hodgkin- and Reed–Sternberg-like cells. Upon EBER in situ hybridization, practically all nuclei were positive. On the upper left, an area of necrosis is seen.

EBV-DLBCL, although by definition composed of large B-cells, can appear in variable patterns in the affected tissues. Polymorphic lesions are characterized by centroblasts, immunoblasts, and/or Hodgkin-like or Reed–Sternberg-like cells in a background infiltrate composed of activated lymphocytes, histiocytes, and plasma cells (Figure 7). In monomorphic types, the tumor cells form sheets indistinguishable from DLBCL, NOS [88,93,94,95]. Angioinvasive and angiodestructive growth, sometimes leading to extensive “geographical” necrosis, are often findings that lead pathologists to search for EBV infection. The immunophenotype is characterized by the expression of pan B-cell markers, and three-fourths of the cases have an ABC-like or non-GCB immunophenotype. The expression of CD30 and PD-L1/PD-L2 is common [86,88]. EBV latency patterns can be I, II, or III with a dominance of EBER reactivity +/− LMP1-positivity (types I and II), and EBNA2 coexpression in 25%, with some geographical variation [86,96].

EBV is thought to play a causal role in the pathogenesis of this entity, and EBV-associated proteins have been shown to enhance or mimic B-cell receptor or CD40 signaling. In addition, the mutational burden of EBV-positive DLBCL has been found to be lower than that of EBV-negative DLBCL, and the presence of EBV may substitute for mutations in driver genes, such as *MYD88* and *CD79A* [97]. NGS analyses have revealed alterations in pathways associated with cellular activation and proliferation, such as *NFKB*, *NOTCH, WNT,* and *JAK/STAT,* and immune escape [97,98] Fabian 36604606. Several genes have been described as exclusively mutated in EBV-DLBCL, such as *CCR6*, *CCR7*, *DAPK1, TNFRSF21*, *CSNK2B,* and *YY1* [97]. PD-L1 overexpression is frequent, with younger patients presenting more frequently with high-level PD-L1+ tumors in comparison with elderly patients [86,92,99].

## 11. Diffuse Large B-Cell Lymphoma Associated with Chronic Inflammation

DLBCL associated with chronic inflammation (CI-DLBCL) is an EBV-associated LBCL confined to the natural body or acquired tissue spaces arising in a setting of chronic inflammation. Pyothorax-associated lymphoma (PAL) is the prototypical form. CI-DLBCL most frequently arises in the pleural cavity, bones, joints, and periarticular tissues [100]. It usually develops after a long latency period (10–35 years) after the onset of chronic infection or implant insertion, such as chronic osteomyelitis, metallic implant or surgical mesh implantation, or chronic venous ulcer of the skin [101,102,103,104]. No immunodeficiency is known in these patients. Similar to Fibrin-associated large B-cell lymphoma (FA-LBCL), CI-DLBCL is thought to be related to local immune modulation by the production of IL10 that fosters immune escape of EBV-infected B-cells [100,105,106,107].

Most cases are composed of centroblasts or immunoblasts, and there may be angiodestructive growth with ensuing massive necrosis. Expression of B-cell markers is typical, but plasmablastic differentiation may occur. CD30 expression and aberrant expression of T-cell-antigens have been noted. EBV type III latency is characteristic [101,102,108].

The GEP of CI-DLBCL (PAL) is characterized by EBV association and down-regulation of HLA class 1 expression [109,110,111]. Complex karyotypes are the rule, and recurrent mutations of *TP53* and deletions of *TNFAIP3* have been recorded [112,113].

## 12. Fibrin-Associated Large B-Cell Lymphoma

Fibrin-associated large B-cell lymphoma (FA-LBCL) had been listed as a variant of CI-DLBCL in WHO-HAEM4R. However, taking into account its characteristic presentation and indolent clinical course, both WHO-HAEM5 and ICC have upgraded it to a definite entity. FA-LBCL is an EBV-associated neoplasm that arises in the setting of chronic fibrin deposition in cysts and pseudocyst cavities, such as in the peri-implant space of breast implants and chronic hematomas, and in intravascular or intracardiac lesions, such as in atrial myxomas or endovascular grafts [114,115,116,117,118]. As in CI-DLBCL, mechanisms leading to local immune impairment are thought to lead to the escape of EBV-positive B-cells from immune surveillance [119].

**Figure 8:**
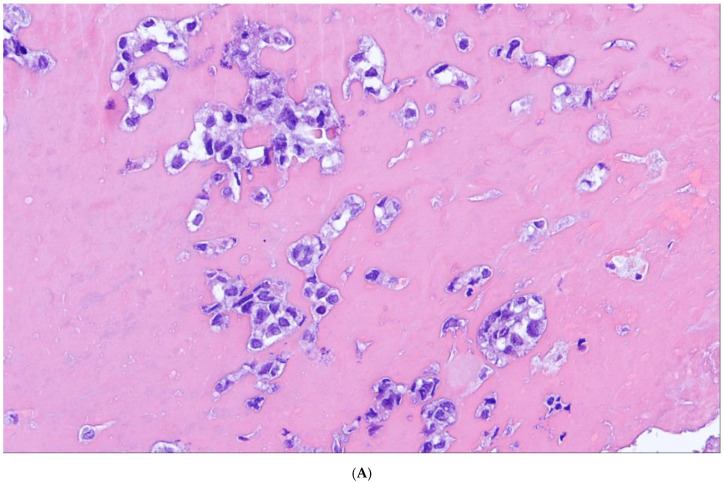

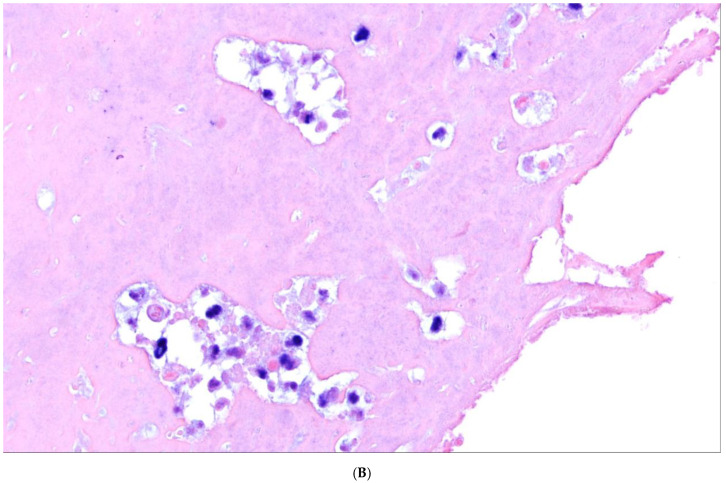
Fibrin-associated large B-cell lymphoma (**A**) (H&E ×200). Large transformed cells are floating in the background of fibrin and cellular debris. (**B**) (×200) EBER in situ hybridization confirmed EBV association.

The typical histological findings are aggregates of large transformed cells floating in the background of fibrin and cellular debris (Figure 8A,B), however, there may be an infiltration of the stroma in cardiac myxomas or capsular tissues as well. Of importance, there is no tumor mass formation and/or invasive growth in FA-LBCL setting this neoplasm apart from EBV-DLBCL and CI-DLBCL. As with CI-DLBCL, aberrant expression of T-cell markers may occur. EBV is present in the vast majority of cases (Figure 7B), but rare EBV-negative FA-DLBCL have been encountered [120,121]. EBV type III latency is the rule [117]. Little is known about the genetic background of FA-LBCL, although occasional cases with *BCL6* or *MYC* translocations have been described.

## 13. Fluid Overload-Associated Large B-Cell Lymphoma

Fluid overload-associated LBCL (FO-LBCL) is a large B-cell lymphoma presenting as serous effusions often in patients with fluid overload states. Of importance, it is not associated with KSHV/HHV8-infection of the tumor cells, hence the name “HHV8- and EBV- negative primary effusion-based lymphoma” in the ICC. Most cases present in the pleural cavity followed by effusions in the pericardium and peritoneum [122]. By definition, no mass formation is detected at presentation but can occur during relapse or disease progression [123].

**Figure 9:**
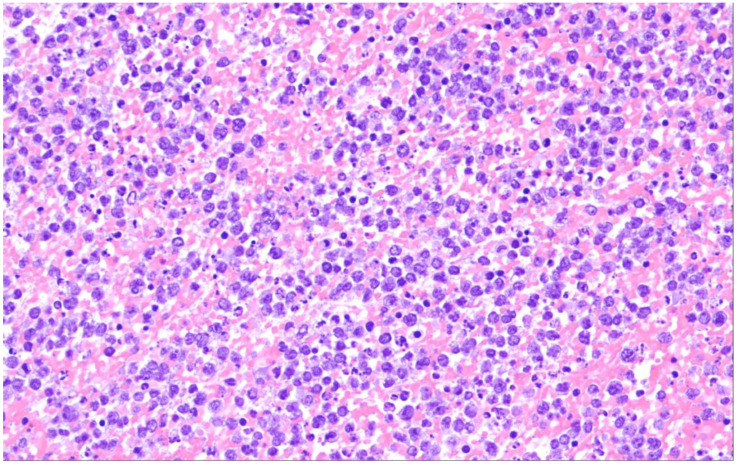
Fluid overload-associated large B-cell lymphoma (H&E ×200) This cytoblock preparation demonstrates massive shedding of large lymphoid cells into the pleural fluid.

The cytological spectrum of the tumor cells as seen in smears or cytoblocks is broad and centroblastic, immunoblastic, or anaplastic cells can be seen (Figure 9). They usually express pan B-cell markers but some of them, in particular, CD20, can be negative. A non-GCB immunophenotype is common, and CD30 expression is variable.

In roughly half of the cases, FO-LBCL is associated with a fluid overload status. KSHV/HHV8 infection is not detected, but a proportion of cases do arise in hepatitis C-positive patients [123,124,125]. EBV association has been reported in 10–30% of cases [123,125]. In the ICC proposal, however, EBV-associated cases are excluded from this entity. The age peak in elderly patients points to immune senescence as a pathologic factor, but overt immunodeficiency by definition is absent. Few genetic data are available pointing to complex copy number changes, recurrent deletions at 3p and 6q, and structural alterations in the *MYC* locus in 50% of cases [124,126]. Mutations have been found in chromatin-modifying genes and *MYD88, IRF4*, and others [126].

## 14. Plasmablastic Lymphoma

**Figure 10:**
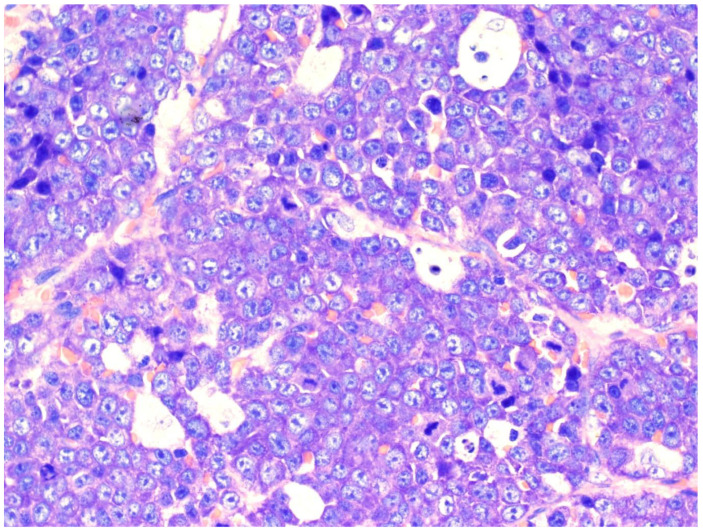
Plasmablastic lymphoma (Giemsa ×400). This photomicrograph shows cohesively arranged immunoblastic/plasmablastic cells with broad deeply basophilic cytoplasm. In this case, CD20 was negative, and both CD138 and MUM1 were strongly expressed.

Plasmablastic lymphoma (PBL), similar to ALK-positive LBCL, is an aggressive lymphoma composed of immunoblastic or plasmablastic cells (Figure 10) that express a plasmablastic immunophenotype and that is negative for CD20 [127,128,129,130]. No major changes have been made from WHO-HAEM4R. PBL most often arises in settings of immune deficiency, most commonly in HIV+ patients and more rarely in patients that have undergone organ transplantation or have been diagnosed with autoimmune disease [127]. In around 30% of patients, however, there is no evidence of immune deficiency [129,130,131,132,133]. Most patients with PBL have an extranodal disease, most commonly, especially in HIV+ patients, in the oral cavity, nasal sinuses, the nasopharynx, and orbit but all extranodal localizations, including the bone marrow, can be involved [134,135].

Translocations involving *MYC*, most often as *IGH::MYC* fusion, have been detected in 50% of cases [136], and EBV infection of the tumor cells is found in 70% of cases [136]. GEP has identified a gene signature similar to plasma cell neoplasms and different from that of DLBCL [137]. More recent NGS studies have identified recurrent mutations in *KRAS* and *NRAS* genes and in genes of the *JAK-STAT* pathway [136,138].

Plasma cell myeloma/multiple myeloma (PCM/MM) with plasmablastic features are a relevant differential diagnosis of PBL because the morphology and the immunophenotype of PBL and PCM/MM are nearly identical and because both *MYC* rearrangements and EBV-association have been noted in plasmablastic PCM/MN; therefore, the clinical presentation is often crucial in rendering the correct diagnosis [139,140,141].

## 15. Primary Large B-Cell Lymphomas of Immune-Privileged Sites

Primary LBCL of immune-privileged sites (IP-LBCL) represents a newly defined entity, or umbrella, in WHO-HAEM5 comprising primary DLBCL that arise in the central nervous system (PCNS-LBCL), the vitreoretina (PVR-LBCL), and the testis (PT-LBCL) in immunocompetent patients. In the ICC proposal, the primary DLBCL of the CNS and the testis continue to be separately listed. DLBCL arising in these localizations have similar morphological, immunophenotypic, and molecular genetic features and have been grouped into one category because of their common background of an origin in immune “sanctuaries” created by precise anatomical structures, such as the blood-brain, blood-retinal and blood-testicular barriers, and specific mechanisms of immune regulation [120,142,143].

**Figure 11:**
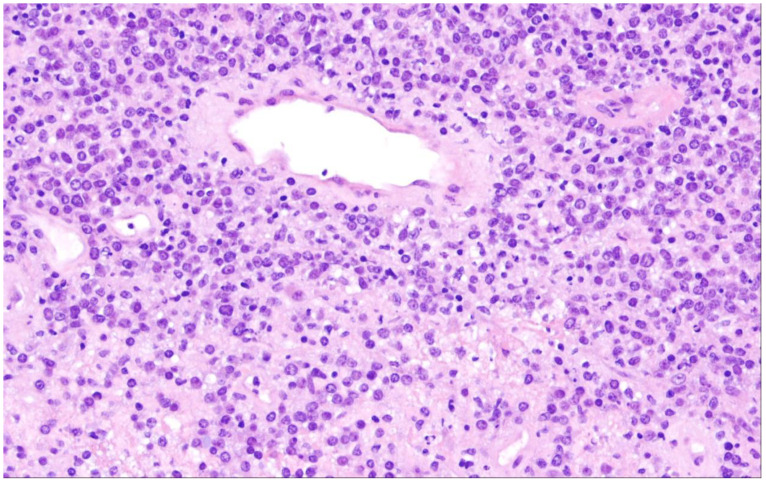
Primary large B-cell lymphomas (LBCL) of immune-privileged sites (H&E ×200). This is an example of a primary central nervous system LBCL with diffuse and perivascular infiltration patterns.

These tumors overall are rare, occurring in middle-aged patients; their clinical features depend on the site of origin. PCNS-LBCL, PVR-LBCL, and PT-LBCL consist of medium-sized to large blasts with round to oval, sometimes pleomorphic, nuclei; light chromatin; and eosinophilic to basophilic cytoplasm. PCNS-LBCL typically infiltrate diffusely and/or in perivascular areas (Figure 11) [144], and PT-LBCL infiltrates are also mainly diffuse. In PVR-LBCL, the neoplastic cells occupy the sub-retinal space and/or the perivascular spaces of the retina with cells often also floating in the vitreous [145]. They show similar immunophenotypes with the expression of mature B-cell markers, BCL2, BCL6, and MUM1, while CD10 expression is rarely seen.

The COO of IP-LBCL is a somatically hypermutated mature GC B-cell with in part biased usage of IGHV genes (especially IGHV4-34) also found in autoimmune disorders [146,147,148,149]. Their genetic alterations are associated with immune escape and/or a down-regulation of immune reactions, for example by genetic inactivation of MHC class I and II and *B2M* genes. *MYD88* and *CD79B* are recurrently mutated [150,151,152,153,154,155,156] so IP-LBCL show features of C5/MCD/MYD88 gene signatures [23,24,25,48,154,157,158]. In addition, IP-LBCL display enhanced BCR and Toll-like signaling, and aberrant somatic hypermutation in genes such as *BCL2, MYC, PAX5,* and *IRF4* are frequently seen [153,159]. Genomic imbalances are frequent, including gains in 18q21 and 9p24.3 and losses in 6q21 and 6p21. *CDKN2A* is frequently inactivated by bi-allelic deletions in 9p21 [154,160,161,162].

## 16. Primary Cutaneous Diffuse Large B-Cell Lymphoma, Leg Type

**Figure 12:**
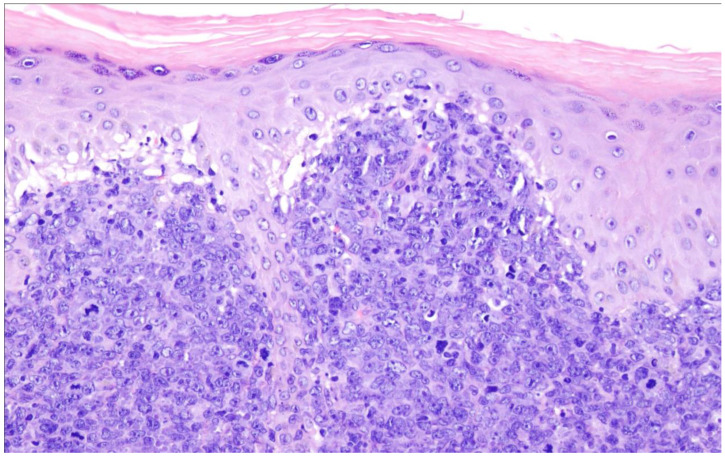
Primary cutaneous diffuse large B-cell lymphoma, leg type (H&E ×200). The large blastic tumor cells are invading the skin and reach the overlying epidermis.

Primary cutaneous diffuse large B-cell lymphoma, leg type (PCLBCL-LT) is an aggressive lymphoma consisting of centroblasts and immunoblasts, usually with an ABC-like or non-GCB-like immunophenotype commonly arising on the lower leg (Figure 12). However, 10–20% of cases have been described at other sites, such as the trunk or the upper extremities [163,164,165,166]. PCLBCL-LT is a disease of typically older patients, with a median age of 75 years, and has a male/female ratio of 1:2-4 [167,168].

Upon histological examination, there is a usually non-epidermotropic infiltration of the dermis and subcutis, consisting of sheets of centroblasts and immunoblasts positive for B-cell markers. Characteristically, there is strong co-expression of BCL2, MUM1, FOXP1, MYC, and cytoplasmic IgM. The proliferation index is high. Aberrant immunophenotypes with the expression of CD10 may be seen in some cases [163,169,170,171,172,173].

PCLBCL-LT shows a high level of IgHV somatic hypermutations. Translocations involving *IGH, MYC,* or *BCL6* frequently occur in 50%, 5–40%, and 25–50%, respectively [166,174,175,176,177,178]. Some cases harbor high-level amplifications in 18q21.3, the site of the *BCL2* locus [166,179]. Recurrent losses have been demonstrated in 9p21 (*CDKN2A* and *CDKN2B*) and for *TNFAIP3* and *PRDM1* [166,179,180]. Mutations in *MYD88* frequently occur in 70% of cases, and PCLBCL-LT also frequently harbors mutations in *CD79B, TNFAIP3, CARD11,* and *PIM1* [166,175,181,182,183,184]. In addition, deletions of HLA loci and *CIITA* as well as rearrangements of *CD274* have been described [166,183]. Of note, the genetic landscape and GEP of PCDLBCL-LT are similar to IP-LBCL of immune-privileged sites, such as those seen in the CNS, the vitreoretina, and the testis.

## 17. Intravascular Large B-Cell Lymphoma

The definition of intravascular LBCL (IVLBCL) was not changed in WHO-HAEM5 or ICC compared to WHO-HAEM4R. IVLBCL is an aggressive extranodal B-cell lymphoma showing a proliferation of large B-cells within the lumens of blood vessels. Different subtypes are recognized according to the leading clinical presentation: the classic subtype with fever, local organ-specific symptoms, and/or multiorgan failure; in the cutaneous subtype, the disease is confined to the skin at presentation, and this subtype is described as less aggressive than the classic subtype. In the haemophagocytic subtype, symptoms related to haemophagocytic syndrome predominate. Generally, IVLBCL is a disease of elderly patients, and the haemophagocytic subtype has been predominantly seen in Asian patients [185,186,187].

**Figure 13:**
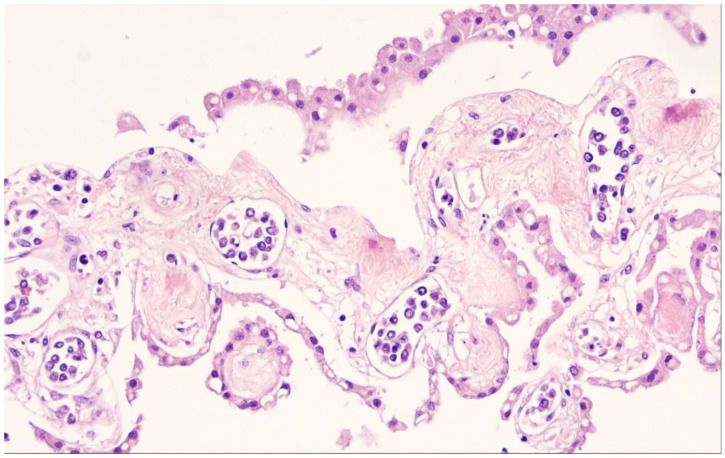
Intravascular large B-cell lymphoma (H&E ×200). The typical blastic tumor cells are seen within distended capillary vessels of the leptomeninx.

In histologic preparations, large B-cells are found within the lumens of capillaries and small vessels with a certain size range (Figure 13). The immunophenotype is characterized by the expression of pan B-cell markers and often with reactivity for CD5 and PD-L1 [188,189]. Most cases have a non-GC B-cell immunophenotype, however, CD10 reactivity has been reported in 10% of cases [190].

Pathogenetically, the lack of expression of adhesion molecules, including integrins and chemokine receptors, prevents vascular adhesion and transvascular migration of the tumor cells [191,192,193,194,195]. Mutations in *MYD88*, *CD79B*, *SETD1B*, and *HLA-B* have been reported [196,197,198,199], and structural alterations in *CD274* (*PD-L1*) and *PDCD1LG2* (*PD-L2*) genes are recurrent [198]. Therefore, IVLBCL, biologically, has some similarities with IP-LBCL.

## 18. Primary Mediastinal Large B-Cell Lymphoma

Primary mediastinal large B-cell lymphoma (PMBCL), as the name implies, arises in the mediastinum, and its founder cell most probably resides in the thymus. PMBCL is a rare lymphoma often affecting adolescents and young adults and has a male-to-female ratio of 1:2. Secondary involvement of the mediastinum by systemic (nodal) lymphoma has to be excluded. Spread outside the mediastinum may occur at relapse [200].

**Figure 14:**
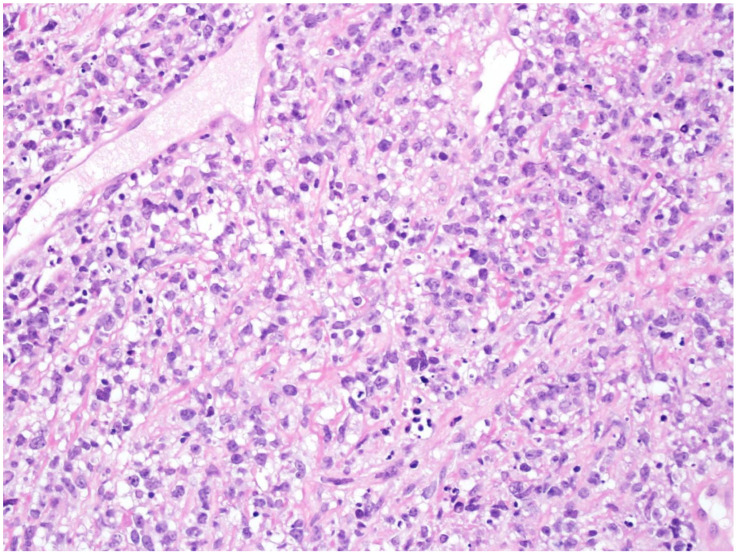
(H&E ×100). In this example, large tumor cells, many of them with a clear cytoplasm, are seen in a background of in part compartmentalizing sclerosis.

The tumor cells forming the characteristic mediastinal mass show a range of cytomorphologies and can resemble centroblasts, immunoblasts, or more pleomorphic, sarcomatoid, or Hodgkin-like cells. They are often associated with sclerosis forming broad bands or leading to fine compartmentalization of the tissue (Figure 14) [201,202,203]. The immunophenotype is characteristic, but not specific; the tumor cells are positive for pan B-cell antigens and frequently also express CD30 and CD23 [204,205], albeit with variable intensity and number of positive cells. Expression of MAL, p63, CD200, PD-L1, and PD-L2 proteins is considered quite specific for PMBCL [205,206,207,208,209,210,211].

The genetic landscape of PMBCL is rather distinctive, and GEP has revealed considerable overlap with classic Hodgkin lymphoma (also frequently arising in the mediastinum) [212,213,214]. Characteristic genetic features are gains in chromosomal band 2p16.1 involving *REL* [215] and amplifications in 9p24.1, the location of *JAK2* and *PD-L1/L2* [216]. In addition, rearrangements in 16p13, harboring *CIITA,* and *PD-L1* and *PD-L2* [217,218] are a characteristic feature of PMBCL. Characteristic point mutations are detected in *SOCS1*, *GNA13*, *STAT6*, *CD58*, *B2M*, *ITKP*, *TNFAIP3,* and *IL4R*. These observations allow the tracing back of proliferation, prevention of apoptosis, and immune evasion to the pathogenetic features of PMBCL.

## 19. Mediastinal Grey Zone Lymphoma

While the definition and characteristic features of PMBCL have remained largely identical in WHO-HAEM4R and WHO-HAEM5, Mediastinal grey zone lymphoma (MGZL), previously designated “B-cell-lymphoma, unclassifiable, with features intermediate between DLBCL and classic Hodgkin lymphoma” in WHO-HAEM4R, has now been renamed both in WHO-HAEM5 and ICC owing to the fact that it mainly arises in the mediastinum. There are two basic types of MGZL: one with the morphology of Hodgkin- and Reed–Sternberg (HRS)-like cells, but the expression of a preserved pan B-cell program, and the second with the morphology of LBCL, but with inappropriate reactivity for antigens typically expressed in HRS-cells, most often for CD15 [219,220,221]. Rare cases with morphologic and immunophenotypic features of MGZL outside of the mediastinum have been described in the literature. Such tumors, however, have been shown to harbor different gene expression profiles and DNA alterations [222]. Hence, WHO-HAEM5 recommends classifying them as DLBCL, NOS.

## 20. High-Grade-B-Cell-Lymphoma, NOS

WHO-HAEM4R had introduced two categories of HGBL, namely “high-grade B-cell lymphoma with *MYC* and *BCL2* and/or *BCL6* rearrangements” and “high-grade B-cell lymphoma, not otherwise specified (NOS)”. As mentioned above, cases with dual rearrangements of *MYC* and *BCL2* and high-grade morphology are now listed in the new entity of DLBCL/HGBL-*MYC/BCL2*. Tumors with an *MYC* and *BCL6* double hit and high-grade or large cell morphology have been designated genetic subtypes of HGBL, NOS or DLBCL, NOS in WHO-HAEM5. According to the new classification, HGBL, NOS is viewed as a heterogeneous category of aggressive B-cell lymphomas composed of medium-sized or blastoid cells (high-grade morphology), which do not fit into other defined entities of aggressive B-cell lymphomas. HGBL, NOS is rare, representing less than 2% of lymphomas.

**Figure 15:**
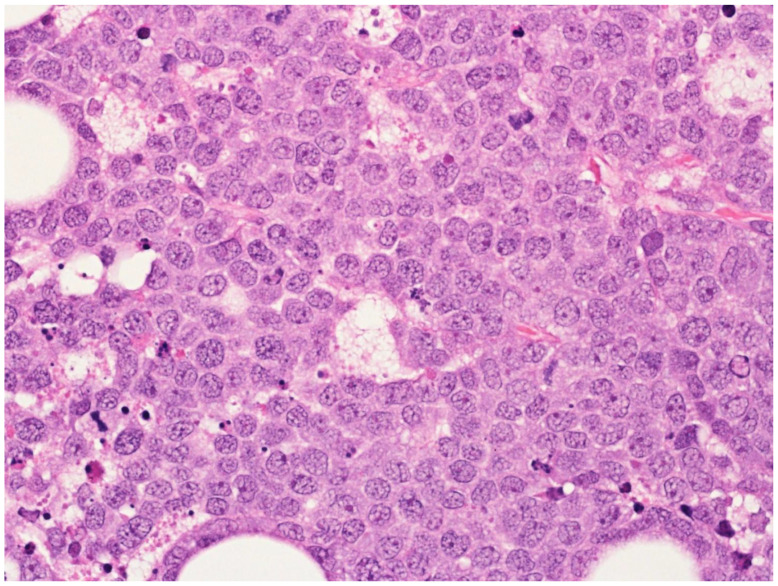
High-grade B-cell lymphoma, NOS (H&E ×400). The tumor cells are small to medium-sized with scant cytoplasm and roundish nuclei. No apparent starry-sky pattern is seen. This tumor was negative for *MYC*, *BCL2*, and *BCL6* rearrangements and also negative for 11q aberrations.

Usually, there is a diffuse growth pattern of moderately cohesive or non-cohesive medium-sized or blastoid cells having variously been described as “Burkitt-like”, intermediate between BL and DLBCL, or blastoid (Figure 15) [223,224,225,226]. However, more recent data indicate that the reproducibility of these categories may be low. The tumor cells express pan-B-cell antigens and have a GCB-like immunophenotype with CD10 and BCL6 expression in the majority of cases and MUM1 in 60% of cases. BCL2 reactivity is seen in 60–70%, and a high proliferation index is typical [227].

In HGBL, NOS, complex karyotypes have been seen in classical cytogenetic analyses and in array-based comparative genomic hybridization (CGH). *MYC* is rearranged in up to 50% of cases, whereas *BCL2* or *BCL6* translocations are rare [36,226,228,229]. NGS analyses have elucidated frequent mutations in *ID3*, *BCL2, TCF3, CCND3, CREBBP, EP300,* and *EZH2*. More recent sequencing data have identified *KMT2D* and *TP53* as the most commonly mutated genes [36]. Using the LymphGen algorithm, HGBL, NOS are grouped into the unclassified category. Of high interest, in GEP, more than 50% of HGBL, NOS harbor the “double hit” signature that had been established from DLBCL with dual *MYC* and *BCL2* rearrangements and that points to dark zone biology [36]. One important differential diagnosis of HGBL, NOS is Burkitt lymphoma (BL), since it shares with HGBL, NOS the presence of medium-sized tumor cells and a GCB immunophenotype in the majority of cases. In addition, *MYC* rearrangements are seen in 50% of HGBL, NOS. However, as a rule, HGBL, NOS shows more pleomorphic or blastoid tumor cells different from those of BL. Of importance, in most cases, HGBL, NOS is also strongly reactive for BCL2, which excludes BL. In cases that do not express BCL2 and that harbor an isolated *MYC* rearrangement, arriving at the correct diagnosis may indeed be difficult and require additional genetic analyses such as finding an absence of an *IG::MYC* fusion or higher genetic complexity in HGBL, NOS [228].

## 21. Diffuse Large B-Cell Lymphomas Arising in Immune Deficiency/Dysregulation

DLBCL arising in the setting of immune deficiency/dysregulation (IDD) are in general histologically indistinguishable from their non-IDD counterparts (DLBCL, NOS) and usually present with diffuse sheets of large B-cells. These lymphomas may be seen in HIV infection, in post-transplant settings, in the setting of autoimmune disease, therapy-related, or in the setting of inborn errors of immunity (IEI). In an era in which novel drugs aimed at immuno-modulation or immunotherapy, e.g., CAR-T, are continuously developed, new types of IDD lesions may emerge, and the frequency and spectrum of IDD-DLBCL may broaden.

Generally, these lymphomas can be DLBCL or plasmablastic in nature, and an ABC-like GEP or non-GCB immunophenotypical constellation is more frequent, especially if associated with EBV infection. The genetic alterations encountered are related to those seen in samples from non-immunocompromised individuals, however, their frequencies may vary. For example, EBV-positive DLBCL have been reported to harbor fewer genetic alterations in the NFkB pathway than their DLBCL, NOS, counterparts [230,231]. In contrast, EBV-negative DLBCL in IDD are genetically more similar to lymphomas in immunocompetent patients and often have a GCB-like GEP [230,231,232,233]. One particular type of DLBCL commonly associated with immunodeficiency is KSHV/HHV8-positive DLBCL, the occurrence of which is usually associated with severe immune deficiency, but may also be seen in non-immunocompromised patients [234,235,236]. The majority of the patients are HIV-positive males.

In some instances, it is difficult, if not impossible, to decide whether a tumor arose in the setting of immunodeficiency or not. One example is lymphomas arising in elderly patients, which has led to the concept of immune senescence as a source of IDD. However, immunodeficiency related to immune senescence cannot be objectively diagnosed so far, and therefore, some tumor classes may be confounded with tumors arising in unrecognized IDD settings. If manifest immune deficiency and/or dysregulation aside from immune senescence is recognized in a patient with aggressive lymphoma, this tumor should be classified as IDD-associated neoplasm.

## 22. Perspective

Considerable progress has been achieved in our knowledge of the molecular pathogenesis of LBCL within the past decade. For most of the different entities, elucidation of the genetic landscape has enabled us to refine the classification of tumors and to define new subgroups. Of importance, our increasing knowledge has significantly contributed to our ability to relate distinct histomorphologic features with the underlying biology that characterizes these neoplasms. Owing to the still preliminary nature of some of these data, and pending agreement on the unification of platforms and algorithms, it would be premature to now coin an entirely molecular subclassification within and among defined entities. We will most probably see this in the upcoming 6th edition of the classification. For this task, however, and the time in between, WHO-HAEM5 provides a robust framework to incorporate these new findings.

## 23. Conclusions and Future Directions

There has been a vast accumulation of new molecular data in the field of the LBCL, allowing us to more precisely define entities and, at the same time, refine their diagnostic criteria. By definition, the classification laid down in WHO-HAEM5 is only a “snapshot in time” and soon, new data will challenge these new definitions and concepts. However, the classification of LBCL and the more sophisticated deciphering of the molecular basis of their origin and progression provides a solid template for future work, ultimately for the sake of patients.

## Figures and Tables

**Table 1:** Large B-Cell Lymphomas in WHO-HAEM5.

Diffuse large B-cell lymphoma, NOS
T-cell/histiocyte-rich large B-cell lymphoma
Diffuse large B-cell lymphoma/high-grade B-cell lymphoma with *MYC* and *BCL2* rearrangements
ALK-positive large B-cell lymphoma
Large B-cell lymphoma with *IRF4* rearrangement
High-grade B-cell lymphoma with 11q aberrations
Lymphomatoid granulomatosis
EBV-positive diffuse large B-cell lymphoma
Diffuse large B-cell lymphoma associated with chronic inflammation
Fibrin-associated large B-cell lymphoma
Fluid overload-associated large B-cell lymphoma
Plasmablastic lymphoma
Primary large B-cell lymphoma of immune-privileged sites
Primary cutaneous diffuse large B-cell lymphoma, leg type
Intravascular large B-cell lymphoma
Primary mediastinal large B-cell lymphoma
Mediastinal grey zone lymphoma
High-grade B-cell lymphoma, NOS

## Data Availability

Not applicable.
